# Advantage of Vital Sign Monitoring Using a Wireless Wearable Device for Predicting Septic Shock in Febrile Patients in the Emergency Department: A Machine Learning-Based Analysis

**DOI:** 10.3390/s22187054

**Published:** 2022-09-17

**Authors:** Arom Choi, Kyungsoo Chung, Sung Phil Chung, Kwanhyung Lee, Heejung Hyun, Ji Hoon Kim

**Affiliations:** 1 Department of Emergency Medicine, Yonsei University College of Medicine, 50 Yonsei-ro, Seodaemun-gu, Seoul 03722, Korea; 2 Division of Pulmonary and Critical Care Medicine, Department of Internal Medicine, Yonsei University College of Medicine, 50 Yonsei-ro, Seodaemun-gu, Seoul 03722, Korea; 3 AITRICS, 28 Hyoryeong-ro 77-gil, Seocho-gu, Seoul 06627, Korea

**Keywords:** deterioration, emergency department, wearable device, septic shock, vital sign monitoring, machine learning

## Abstract

Intermittent manual measurement of vital signs may not rapidly predict sepsis development in febrile patients admitted to the emergency department (ED). We aimed to evaluate the predictive performance of a wireless monitoring device that continuously measures heart rate (HR) and respiratory rate (RR) and a machine learning analysis in febrile but stable patients in the ED. We analysed 468 patients (age, ≥18 years; training set, n = 277; validation set, n = 93; test set, n = 98) having fever (temperature >38 °C) and admitted to the isolation care unit of the ED. The AUROC of the fragmented model with device data was 0.858 (95% confidence interval [CI], 0.809–0.908), and that with manual data was 0.841 (95% CI, 0.789–0.893). The AUROC of the accumulated model with device data was 0.861 (95% CI, 0.811–0.910), and that with manual data was 0.853 (95% CI, 0.803–0.903). Fragmented and accumulated models with device data detected clinical deterioration in febrile patients at risk of septic shock 9 h and 5 h 30 min earlier, respectively, than those with manual data. Continuous vital sign monitoring using a wearable device could accurately predict clinical deterioration and reduce the time to recognise potential clinical deterioration in stable ED patients with fever.

## 1. Introduction

Patients who visit the emergency department (ED) for fever account for 5% of all patients and 15% of all older adult patients in the ED, and those with chronic diseases or aged ≥65 years have a 30-day mortality rate of 7–9% [1]. Sepsis is a serious complication in patients who present to the ED with febrile symptoms and can lead to a fatal clinical outcome [2]. Therefore, it is crucial to recognise the deterioration in these patients at the early stages of their disease courses and to promptly intervene to avoid poor outcomes [3,4]. Since changes in vital signs are important indicators of physiological decline, their immediate detection enables early recognition of clinical deterioration and intervention [5,6,7,8]. However, it is practically impossible for physicians to closely monitor and observe each patient who presents to the ED. Therefore, close patient monitoring is prioritised for patients who are unstable during triage or the first check-up. Accordingly, early detection of clinical deterioration may not be achieved by intermittent measurement of vital signs since resources are not continuously provided to patients initially deemed as not requiring close monitoring [9,10,11,12,13,14,15]. Furthermore, febrile patients have become more vulnerable to deterioration since the outbreak of the coronavirus disease 2019 (COVID-19) as bedside patient observation has been restricted to control the spread of the disease [16,17].

Recently, wireless wearable devices for continuous monitoring of heart rate (HR), respiratory rate (RR), body temperature, and patient location have been introduced worldwide. Previous studies found that these devices can improve the safety of patients who have difficulty accessing regular monitoring devices [18,19,20,21]. However, the reliability of these devices in relevant clinical environments has not been fully validated; thus, their practical use in clinical settings is limited [22,23,24]. Furthermore, only a few studies were conducted in the ED, and most studies focused on the use of these devices in patients admitted to wards [15,25].

Due to the tremendous advances in the development of machine learning and deep learning algorithms, the availability of large databases, and the increase in computational processing power, machine learning in the medical field has rapidly evolved over the past two decades, with a wide range of applications using several different algorithms such as natural language processing, data mining, clustering, and classification [26].

The size of data obtained using a continuous monitoring device is much larger than that obtained using the manual method in which the measurements are obtained intermittently. Machine learning-based analysis is being used in various clinical research studies that include large amounts of data since it is suitable to promptly manage large-scale datasets with various datatypes within a short time [27,28].

Although previous studies revealed that frequently monitored vital signs are useful for clinical decision making, the particular type and frequency of vital signs that should be monitored and the correlation between each parameter and poor outcomes are yet to be studied [29]. With the growing number of patients presenting to EDs and the enormous amount of data related to patient monitoring, conventional techniques are considered inadequate to process these data. Therefore, the efficiency of emergency medical practice can be maximised if a large amount of data generated during the emergency department stay can be immediately processed using a machine learning algorithm [30,31].

Therefore, in the present study, we aimed to evaluate the predictive performance of a wireless monitoring device for the continuous measurement of HR and RR in febrile but stable patients in the ED using machine learning-based analysis.

## 2. Materials and Methods

### 2.1. Study Design and Population

This was a retrospective study conducted using data collected prospectively at the ED of a tertiary teaching hospital from 20 July 2020 to 15 June 2021. We used an interventional design, which involved continuous monitoring of the signals of patients obtained using a wireless wearable device and intermittent manual measurements of the vital signs.

Patients aged ≥18 years who were stable but had a body temperature over 38 °C upon arrival and were admitted to the isolation care unit in the ED were enrolled in this study. The isolation care unit in the ED is for patients with suspected COVID-19. All patients who met the enrolment criteria were asked to participate in the study on arrival at the ED, and written informed consent was obtained if the patient agreed to participate in the study. This study was approved by the Institutional Review Board of Yonsei University College of Medicine, Severance Hospital (no. 1-2019-0058).

### 2.2. Description of the Wireless Wearable Device

Hicardi (MEZOO Co., Ltd., Wonju-si, Gangwon-do, Korea) is an 8 g, 42 × 30 × 7 mm, wireless, and wearable adhesive monitoring device certified as a medical device by the Ministry of Food and Drug Safety of Korea. This device is used to monitor and record single-lead electrocardiograms (ECGs), RR, and skin surface temperature, as well as patient location and activity (Figure S1). It comprises a reusable sensor module and a disposable adhesive patch that houses a two-point ECG electrode. The reusable sensor module contains a microprocessor, an ECG/respiration circuit, a temperature sensor, a triaxial accelerometer, a Bluetooth low-energy (BLE) transceiver, and a 50 mAh Li-ion polymer battery. This device can be attached to the chest and can continuously measure signals for up to 24 h. The sensor processes and transmits the signals through BLE to a mobile device with a smartphone application. This application can display ECG, HR, RR, patient posture, and geographical location in real time and transmit the data to a cloud-based monitoring server with a randomised research number. Patient information is not recorded on the mobile device to ensure anonymisation. This system complied with the international standards and guidelines of regulatory authorities regarding cybersecurity [32,33].

The ECG signal is recorded at a rate of 250 samples/s in 14 bits. The algorithm, which calculates the HR using ECG, is based on the automated detection of QRS complexes of ECG waveforms. RR is derived from the changes in respiration detected through the measurement of the electrical impedance of the patient’s thorax. This device with a real-time heartbeat detection algorithm showed 99.95% of the sensitivity, 99.95% of the positive predictivity, and 0.10% of the detection fail rate on the four different databases in the previous study [34].

### 2.3. Data Acquisition

ECG rhythm and respiratory signals were continuously monitored using the wearable wireless device and manually measured intermittently. After obtaining informed consent from the patient, the wireless wearable monitoring device was attached to the patient’s left sternal border. Measurement of vital signs, such as systolic blood pressure, diastolic blood pressure, HR, RR, and mental status, was performed manually and recorded every hour, from baseline to 6 h later, while the wearable device was attached to the patient. If the patient died, was discharged, or was hospitalised within 6 h from the time of enrolment in this study, the data collected up to that time were used.

The signals were uploaded to the cloud-based server according to the device number and analysed after data collection was completed. The data from the device were separated, encrypted, and stored in electronic medical records in the hospital legacy system [35]. The researchers checked the central monitor and verified the quality of the signal data during data collection to determine whether the ECG and respiratory waveforms were properly transmitted from the mobile device to the cloud server. Regarding the signal data from the wearable monitoring device, 250 Hz ECG waveform data with information about R-peak location and 25 Hz respiratory waveform data were used.

### 2.4. Signal Data Filtering Process

The raw data transmitted by the wearable device were obtained, and invalid data were removed to retrieve continuous vectors of vital signals. Vital sign data were sampled once per second with their timestamps. Artifacts were not removed before the signal data filtering process. For ECG data, one unit was set and sliced to process the outlier so that the QRS wave was at the centre of each unit. If a unit was 1.5 times longer than the median of the units, or if the maximum or minimum values of the units were twice as large or small as the other units, these units were excluded to minimise the noise of the signal. The median HR for each unit was calculated from the entire ECG data, and the units excluded as described above were processed using the carry forward method. For respiratory signal data, the units excluded from ECG data filtering were removed at first, and the number of sections between each peak of the respiratory signal wave was calculated to obtain the RR [36]. If the value of the respiratory wave was 0.5 times smaller or 1.5 times larger than the previous respiratory value, these units were excluded. The median RR for each unit was calculated from the entire respiratory data, and the units excluded as described above were processed using the carry forward method.

### 2.5. Development of the Prediction Model

We processed and utilised 14 different values for model development. Data regarding age, sex, body temperature, and oxygen saturation were recorded on arrival and included as the static values for model development (four values), and data regarding systolic blood pressure, diastolic blood pressure, HR, RR, and mental status obtained every hour were included as the dynamic values (five values); the data were measured either manually or using the wearable device. We then calculated the difference between the data measured hourly and the vital sign data measured initially (five values).

With these data, we created a data fragment to build two different models—a simple computed model that predicted outcomes using only one data fragment in a specific time unit, and the accumulated model that predicted outcomes by considering all previous data fragments comprehensively. For data fragmentation, data units of the 14 previously mentioned values (five static values, four dynamic values, and four difference values), measured every hour, were used as manual data, whereas data units of the same 14 values, with HR and RR replaced with signal data from the wearable device, measured every 5 min, were used as device data.

**Figure 1:**
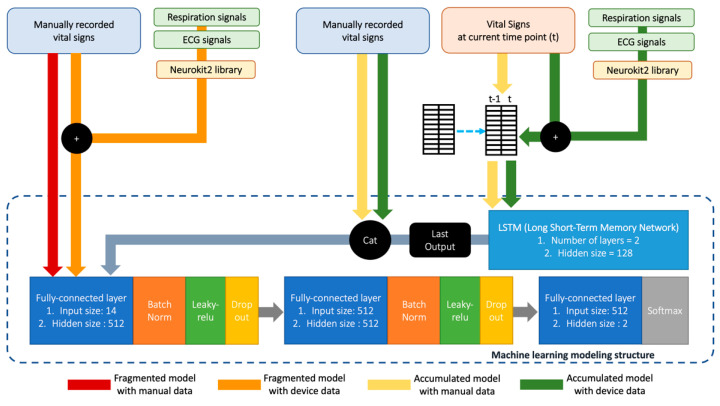
Technical structure of the prediction modelling process.

For the development of the model, we used a convolutional neural network (CNN) and a long-short term memory (LSTM) network for time-sensitive analysis. A CNN is a type of artificial neural network commonly used for the analysis of visual images, such as ECG signals [37]. LSTM is a structure with an artificial recurrent neural network that is used in deep learning algorithms. Compared with standard neural networks, LSTM is applied during unsegmented data handling owing to the property that processes entire sequences of data using feedback connections [38]. The technical structure of the model is shown in Figure 1 and Supplementary Table S1. Considering the average time to evaluate, diagnose, and decide disposition during the ED stay, we set the data acquisition time to 6 h to predict septic shock in febrile patients. Regarding the estimation of the time to predict deterioration, it was assumed and recorded that it took over 6 h to predict septic shock if the model failed to predict it within the first 6 h.

### 2.6. Outcomes

The primary outcome was clinically identified septic shock requiring treatment with vasopressors to maintain a mean arterial pressure of 65 mm Hg or more in the absence of hypovolemia after adequate fluid administration within 24 h from the time of enrolment [39].

### 2.7. Statistical Analysis

Statistical analyses were performed using R software version 3.4.4 for Windows (R foundation for statistical computing, Vienna, Austria). The results are presented as mean ± standard deviation (SD) or median (interquartile range) for continuous variables and as frequencies (%) for categorical variables. The patients were randomly allocated to a training set, a validation set, and a test set in a 6:2:2 ratio for machine learning and model development. The sensitivity and specificity of the models were examined. The discriminative ability of each model was assessed using the area under the receiver operating characteristic curve (AUROC) and the area under the precision–recall curve with 95% confidence interval (CI) values.

## 3. Results

### 3.1. Baseline Characteristics

**Figure 2:**
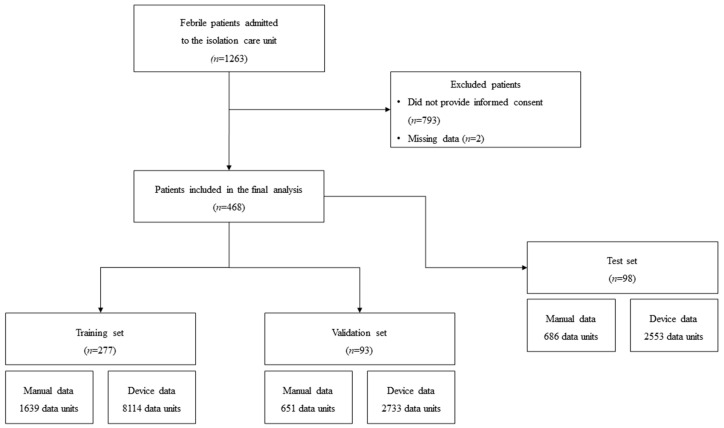
Flowchart of patient selection for the study.

A total of 1263 febrile patients were admitted to the isolation care unit of the ED for COVID-19 screening. We excluded 793 patients who did not provide informed consent. Two patients had missing wearable device data owing to technical issues. Thus, the data of 468 patients, comprising 277 patients in the training set, 93 in the validation set, and 98 in the test set, were eligible for analysis (Figure 2).

Table 1 shows the baseline characteristics of the enrolled patients.

### 3.2. Reliability of the Wireless Monitoring Device

**Figure 3:**
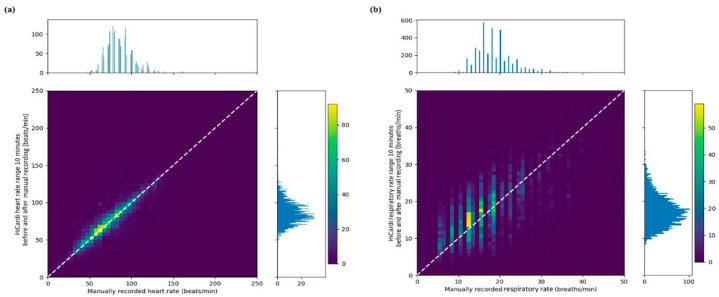
Agreement between the measurements from the wireless monitoring device and manual measurements. (**a**) Agreement between heart rate measurements. (**b**) Agreement between respiratory rate measurements.

Figure 3a shows the agreement between HR data measured using the wireless device and a standard monitoring device (reference standard). The mean difference in HR was −0.685 bpm, with a 95% level of agreement (−15.978–14.607 bpm). Figure 3b shows the agreement between RR data measured using the wireless device and a wall monitor. The mean difference was 1.039 bpm, with a high level of agreement (−5.884–7.963). Bland–Altman plots to compare the Hicardi with a standard measuring method are depicted in Supplementary Figure S2.

### 3.3. Predictive Performance

Of the 468 enrolled patients, 76 (16.2%) suffering from sepsis needed vasopressors to maintain at least 65 mmHg of mean arterial pressure. In the test set, the clinical condition of 16 of the 98 (16.3%) patients deteriorated, resulting in septic shock. The AUROC value of the fragmented model developed using device data was 0.858 (95% CI, 0.809–0.908), which was higher than that developed using manually recorded data (AUROC, 0.841; 95% CI, 0.789–0.893). The accumulated model developed using device data showed better predictive performance (AUROC, 0.861; 95% CI, 0.811–0.910) than that developed using manually recorded data (AUROC, 0.853; 95% CI, 0.803–0.903) (Table 2).

### 3.4. Time to Predict Deterioration

**Figure 4:**
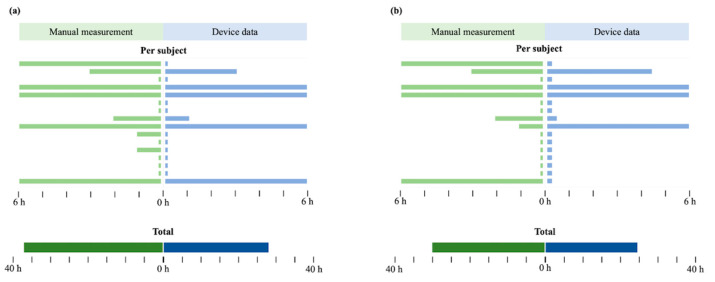
Prediction timepoint data for all patients with positive outcomes. (**a**) Fragmented model. (**b**) Accumulated model.

In the test set, both fragmented and accumulated models developed using device data accurately predicted septic shock within 6 h in one more patient than those developed using manual data. Regarding the time to predict septic shock when the threshold was set at maximum sensitivity with specificity over 0.9, compared with the model with manual data, the fragmented model with device data predicted septic shock at least 9 h earlier in total, and the accumulated model with device data predicted septic shock 5 h 30 min earlier at the minimum. The prediction time point data for all patients with positive outcomes are shown in Figure 4.

### 3.5. Feature Importance Scores of the Models

**Figure 5:**
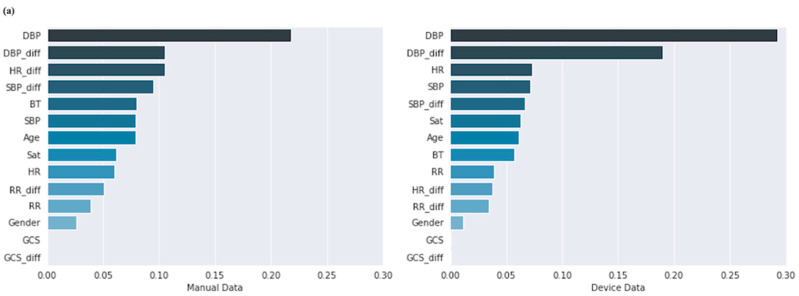

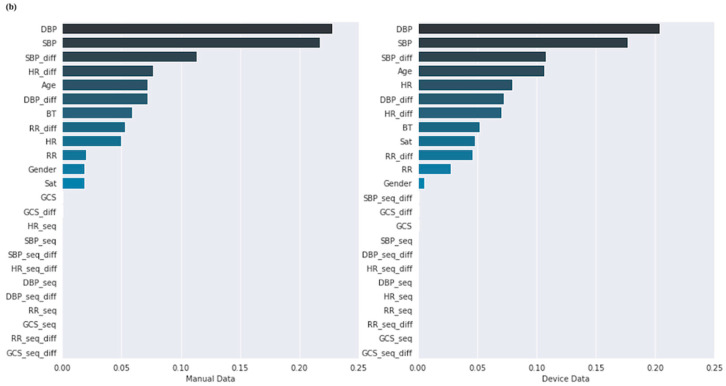
Feature importance scores of the models. (**a**) Fragmented model with manual data (left), with device data (right). (**b**) Accumulated model with manual data (left), with device data (right). SBP, systolic blood pressure; DBP, diastolic blood pressure, HR, heart rate; RR, respiratory rate; GCS, Glasgow coma scale; BT, body temperature; Sat, oxygen saturation; diff, difference; seq, sequence.

As a result, blood pressure (SBP, DBP), both the value of the current time point and the difference value between the present and the time of the visit, showed high feature importance scores in all four models. Additionally, accumulating the latest HR value was important for prediction since the current HR ranked higher in the model using the device data. On the other hand, in the model using manual data, the HR difference value ranked higher. The ranking of features with high importance scores showed a similar tendency in the accumulated and fragmented models. The feature importance scores of the models are shown in Figure 5.

## 4. Discussion

Patients in the ED who show signs of instability during the triage stage are assigned to a treatment area with a standard multi-parameter monitoring device used in intensive care units (ICU) and operating rooms [17]. Since the supply of resources in the ED is limited, it is not possible to assign all patients in the ED to this critical treatment area or to provide a standard monitoring device for each patient. In addition, unlike in the ICU, ED physicians cannot continuously manage the admitted patients for an extended period. Moreover, since patients in the ED are frequently moved out of the ED for radiologic evaluation or urgent procedures, the use of standard monitoring devices with limited mobility in the ED is not practical [40,41]. Additionally, patients in the ED are more likely to become unstable than patients in the general ward, even if their initial vital signs are stable; thus, frequent monitoring of patients in the ED is important. Currently, numerous wireless monitoring devices capable of continuously measuring vital signs have been introduced to compensate for the lack of mobility of standard monitoring devices [42,43]. According to the findings of previous studies, the performances of these devices are not inferior to those of standard monitors; in particular, the measurement of HR using wireless devices has been considerably reliable [22,23]. However, studies that assess the clinical utility of these devices in specific medical settings are rare [44]. Furthermore, several studies conducted to confirm the improvement in clinical outcomes with the use of wireless monitoring devices could not clearly demonstrate the superiority of such devices over the manual measurement of vital signs [24]. The present study demonstrated that continuous measurement of HR and RR using a wireless monitoring device enabled the accurate prediction of clinical deterioration and could reduce the time to predict septic shock in actual clinical practice. In addition, we minimised the contamination of the prediction modelling by the noise that inevitably occurred when using the real-time data by developing an algorithm after pre-processing using time series data processing technology [36]. It is known that clinical deterioration of patients can be predicted more precisely when vital signs are measured and recorded frequently and the changes at intervals are considered comprehensively [45,46,47,48,49]. The predictive model developed in the present study confirmed that even though only two vital signs were measured continuously, the predictive value, except sensitivity for clinical deterioration, was increased compared with that of the model developed using intermittent input. In summary, the present study quantitatively confirms the clinical hypothesis that physicians should make a medical decision considering changes in the patient’s condition by acquiring vital sign values more frequently.

Assessment of the risk of potential clinical deterioration in patients who are clinically stable on arrival to the ED is a crucial and challenging task for ED physicians [3,50,51]. In the frequently overcrowded ED, it is practically impossible for medical staff to continuously collect signal data of vital signs manually and use them in real time for the timely detection of clinical deterioration of patients. In addition, the utility of the wearable device can be guaranteed only when there is an algorithm that can accurately predict deterioration by rapidly processing real-time unstructured data generated from the device. Therefore, advanced technologies, such as wearable devices for patient monitoring and the machine learning analysis introduced in this study, can support the complex decision-making task of physicians in clinical settings through the quantification of atypical data, which is currently missing in practice.

Advanced devices that can facilitate patient monitoring have been introduced in previous studies [18,19,20,21]. However, the medical device itself does not change the clinical environment. These technologies can be applied in clinical practice only when the clinical benefit of the device’s performance is clearly identified in a specific clinical scenario [52]. The scenario of the present study meets the needs of ED physicians in clinical practice. Specifically, bedside evaluation of febrile patients who visit the ED has been limited since the outbreak of COVID-19 [16,17]. Accordingly, we utilised a real-world scenario to verify the clinical usefulness of this wireless monitoring device by improving the blind spots in monitoring that may occur owing to the limitations in bedside access by medical staff. In addition, the structure of the prediction model developed was designed to reflect the physician’s point of view and predict clinical deterioration in febrile patients. Since vital signs reflect core information that identifies patients progressing to septic shock [53,54], we used serial values as input information for the model. In particular, the machine learning algorithm used for detecting the earliest time to recognise deterioration was trained to maximise sensitivity in predicting poor outcomes while maintaining high specificity. This was performed considering the characteristics of the ED, where it is critical to not miss deteriorating patients and to initiate treatment within a short period.

The present study had some limitations that should be considered when interpreting its results. Firstly, of the vital sign indicators, only the continuous signal data for HR and RR were used for analysis. The performance of the predictive model can be improved when the continuous signal data of additional indicators are obtained. Secondly, although the data were collected prospectively, the analysis was performed retrospectively; thus, the predictive model did not lead to physician intervention in clinical practice. Therefore, future prospective research is required to confirm whether our predictive model supports the clinical decisions of ED physicians and improves clinical outcomes.

## 5. Conclusions

The present study is a pilot trial in which a wearable device continuously captured patients’ vital signs that occurred in the clinical field but could not be detected and converted into a useful data source for clinical practice. It showed that continuous monitoring of vital signs using a wearable device could predict clinical deterioration accurately and reduce the time to recognise potential clinical deterioration in stable ED patients with fever. Our results support the application of a wearable device in clinical settings to decrease safety risks due to limited ED resources. Similar studies are needed to secure the use of these digital technologies in various clinical settings in the future.

## Figures and Tables

**Table 1:** Baseline characteristics of the enrolled patients.

Characteristics	Value
sex (male)	202 (44.20)
age (years)	56.87 ± 18.80
duration of device use (min)	300.27 ± 100.03
vital signs on arrival	
systolic blood pressure (mmHg)	127.36 ± 22.06
diastolic blood pressure (mmHg)	75.72 ± 11.46
heart rate (bpm)	108.59 ± 18.52
respiratory rate (bpm)	18.16 ± 2.71
body temperature (°C)	38.64 ± 0.57
oxygen saturation (%)	96.77 ± 2.47
Glasgow Coma Scale score	14.98 ± 0.23
use of vasopressors	85 (18.60)

Values are expressed as number (%) or mean ± standard deviation.

**Table 2:** Predictive performance of each model.

Model	Data	AUROC (95% CI)	AUPRC (95% CI)	Sensitivity (95% CI)	Specificity (95% CI)
fragmented model	manual data	0.841 (0.789–0.893)	0.699 (0.598–0.783)	0.731 (0.633–0.811)	0.836 (0.796–0.870)
	device data	0.858 (0.809–0.908)	0.761 (0.664–0.837)	0.710 (0.611–0.792)	0.936 (0.907–0.956)
accumulated model	manual data	0.853 (0.803–0.903)	0.679 (0.578–0.766)	0.710 (0.611–0.792)	0.841 (0.802–0.874)
	device data	0.861 (0.811–0.910)	0.689 (0.588–0.775)	0.699 (0.599–0.783)	0.880 (0.844–0.908)

AUROC, area under the receiver operating characteristic curve; AUPRC, area under the precision–recall curve; CI, confidence interval.

## Data Availability

The data presented in this study are available on request from the corresponding author. The data are not publicly available due to the Personal Information Protection Act in Korea.

## Supplementary Materials

The following supporting information can be downloaded at: https://www.mdpi.com/article/10.3390/s22187054/s1, Table S1: Technical structure of the prediction modelling; Figure S1: The wireless wearable monitoring device and the smartphone application used in the study; Figure S2: Bland–Altman plots to compare the Hicardi with a standard measuring method.
