# Supplementary material for: Advantage of Vital Sign Monitoring Using a Wireless Wearable Device for Predicting Septic Shock in Febrile Patients in the Emergency Department: A Machine Learning-Based Analysis
Source: Sensors (Basel). 2022 Sep 17;22(18):7054. doi: 10.3390/s22187054 (PMC9504566; doi:10.3390/s22187054)
Supplement: Supplementary file 1 [file sensors-22-07054-s001.zip › Supplementary table S1.pdf]

Supplementary table S1. The structure of the models

	Description	Model Structure	Hidden Layer Size	Epochs	Slice Length (sec)	AUROC
**Fragmented model (Manual Data)**	Final Model	Dense + Batchnorm + l-relu +Dropout	512	400		0.841
	No Dropout	Dense + Batchnorm + l-relu	512	400		0.822
	Hidden-Size=256	Dense + Batchnorm + l-relu +Dropout	256	400		0.815
	Hidden-Size=128	Dense + Batchnorm + l-relu +Dropout	128	400		0.778
	Epoch 100	Dense + Batchnorm + l-relu +Dropout	512	100		0.806
	Epoch 200	Dense + Batchnorm + l-relu +Dropout	512	200		0.808
**Fragmented model (Device Data)**	Final Model	Dense + Batchnorm + l-relu +Dropout	512	400	600	0.858
	No Dropout	Dense + Batchnorm + l-relu	512	400	600	0.832
	Hidden-Size=256	Dense + Batchnorm + l-relu +Dropout	256	400	600	0.827
	Hidden-Size=128	Dense + Batchnorm + l-relu +Dropout	128	400	600	0.785
	Model with shorter Slice	Dense + Batchnorm + l-relu +Dropout	512	400	300	0.839
	Epoch 200	Dense + Batchnorm + l-relu +Dropout	512	200	600	0.848
**Accumulated model (Manual Data)**	Final Model	LSTM + Dense + Batchnorm + l-relu +Dropout	Dense: 512 / LSTM: 128	400		0.853
	No Dropout	LSTM + Dense + Batchnorm + l-relu	Dense: 512 / LSTM: 128	400		0.818
	Hidden-Size=256	LSTM + Dense + Batchnorm + l-relu +Dropout	Dense: 256 / LSTM: 128	400		0.82
	Epoch 150	LSTM + Dense + Batchnorm + l-relu +Dropout	Dense: 512 / LSTM: 128	150		0.833
	Epoch 200	LSTM + Dense + Batchnorm + l-relu +Dropout	Dense: 512 / LSTM: 128	200		0.838
**Accumulated model (Device Data)**	Final Model	LSTM + Dense + Batchnorm + l-relu +Dropout	Dense: 512 / LSTM: 128	400	600	0.861
	No Dropout	LSTM + Dense + Batchnorm + l-relu	Dense: 512 / LSTM: 128	400	600	0.845
	Hidden-Size=256	LSTM + Dense + Batchnorm + l-relu +Dropout	Dense: 256 / LSTM: 128	400	600	0.784
	Model with shorter Slice	LSTM + Dense + Batchnorm + l-relu +Dropout	Dense: 512 / LSTM: 128	400	300	0.809
	Epoch 200	LSTM + Dense + Batchnorm + l-relu +Dropout	Dense: 512 / LSTM: 128	200	600	0.85

AUROC, Area under the receiver operating characteristic curve; LSTM, Long short-term memory
