# Supplementary figures and images for: Advantage of Vital Sign Monitoring Using a Wireless Wearable Device for Predicting Septic Shock in Febrile Patients in the Emergency Department: A Machine Learning-Based Analysis
Source: Sensors (Basel). 2022 Sep 17;22(18):7054. doi: 10.3390/s22187054 (PMC9504566; doi:10.3390/s22187054)

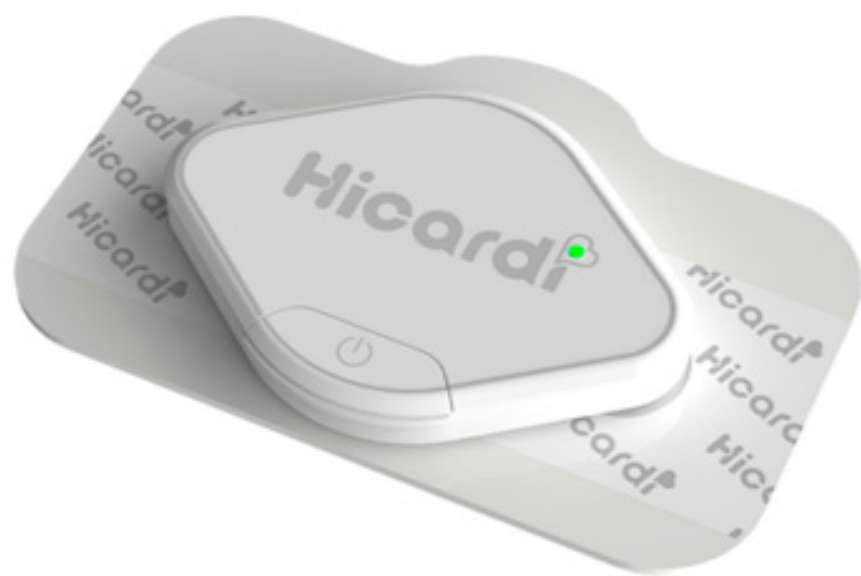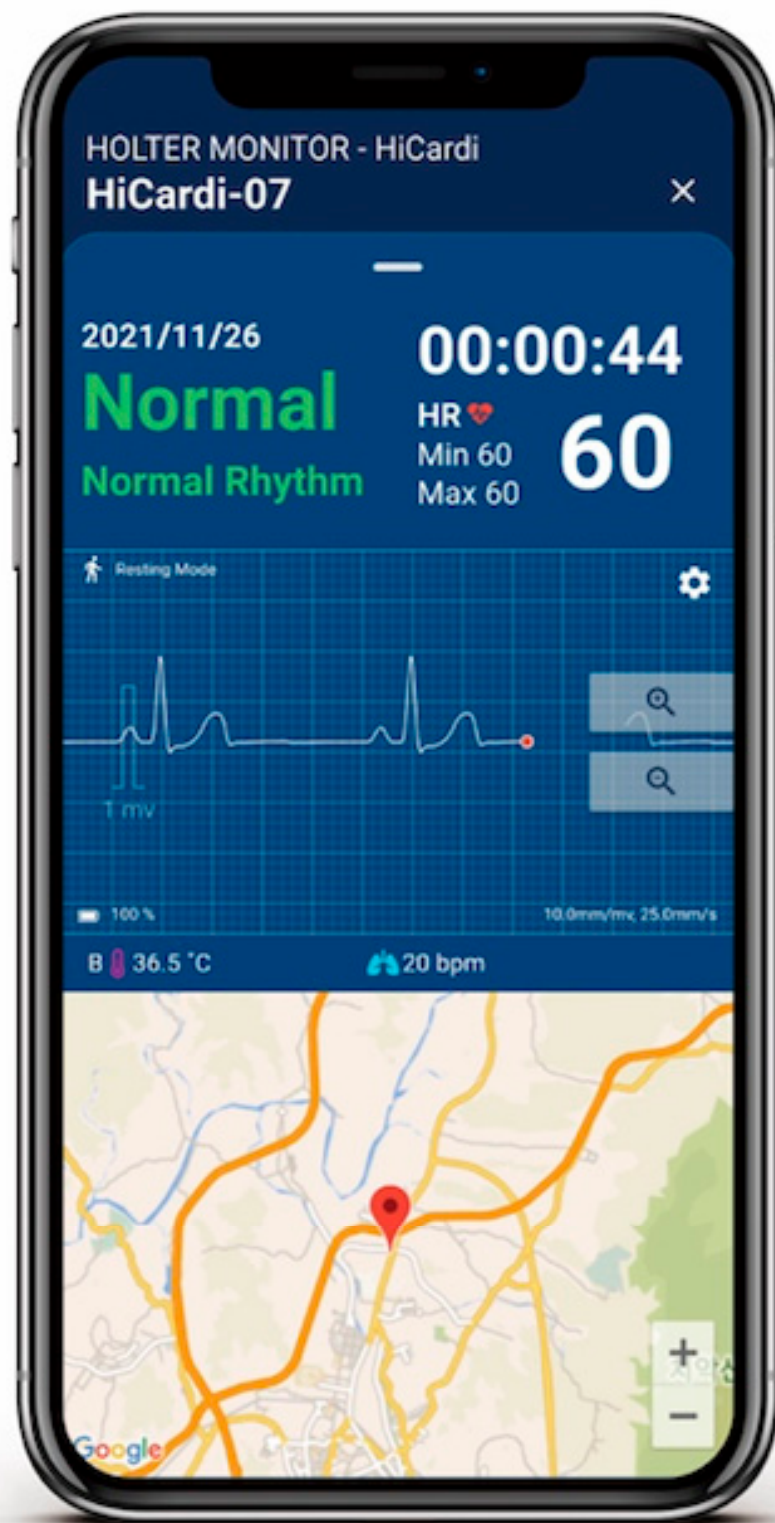

Supplement: Supplementary file 1 [file sensors-22-07054-s001.zip › Supplementary figure S1.pdf]

(a)

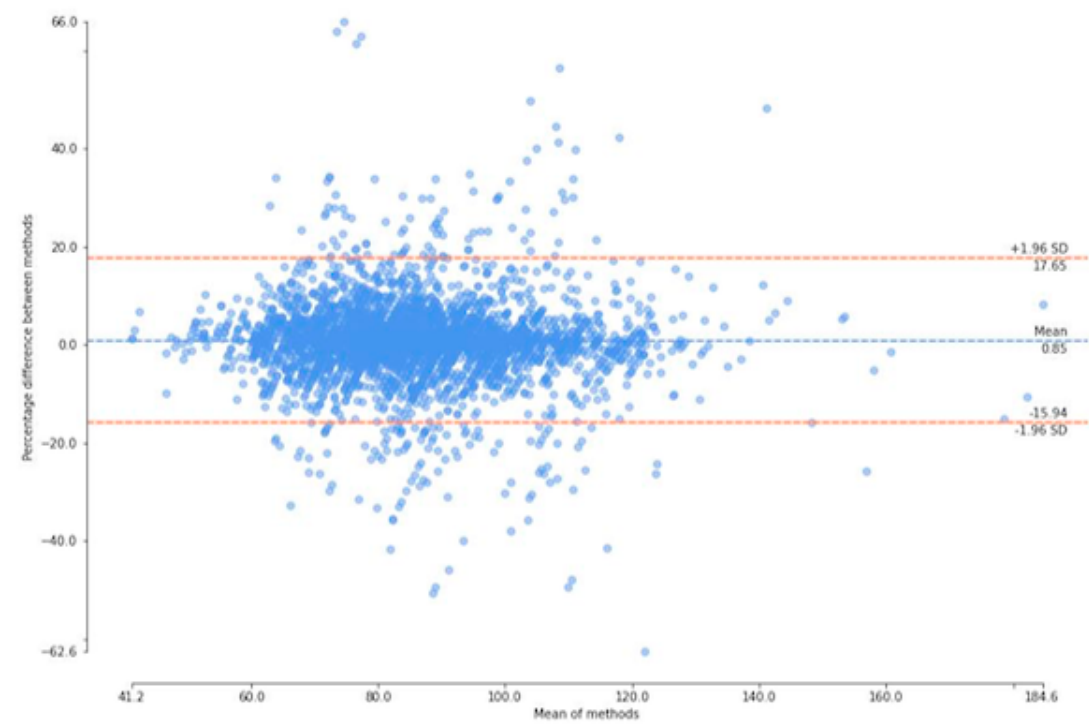

(b)

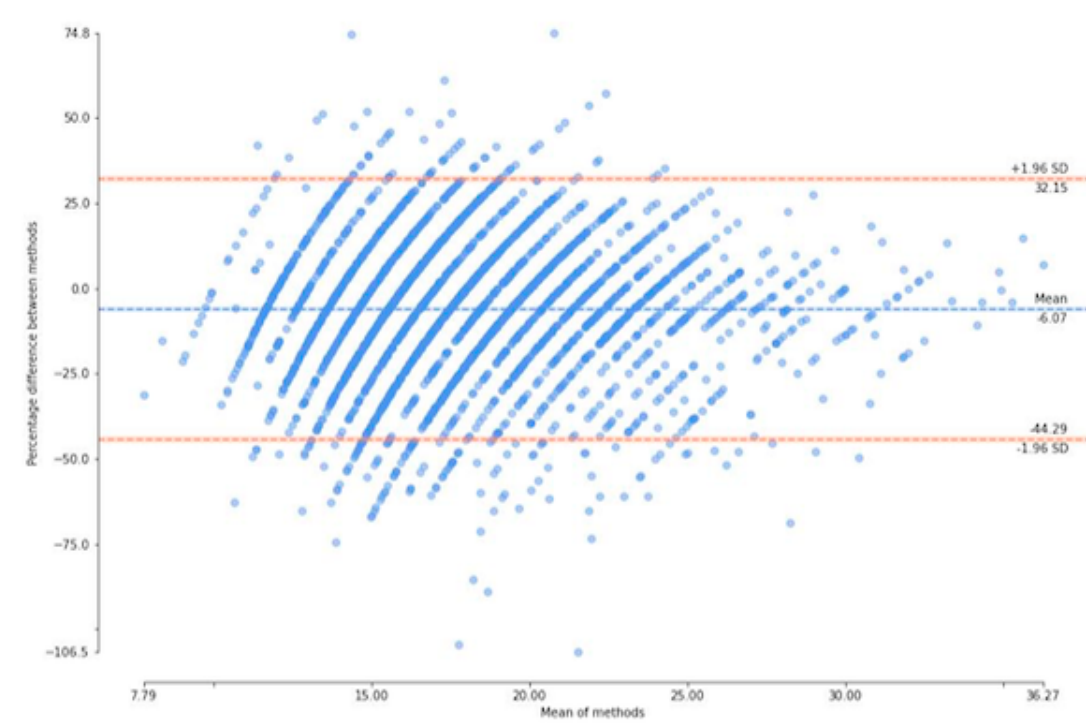

Supplement: Supplementary file 1 [file sensors-22-07054-s001.zip › Supplementary figure S2.pdf]
